# Temporal Differentiation of Crop Growth as One of the Drivers of Intercropping Yield Advantage

**DOI:** 10.1038/s41598-018-21414-w

**Published:** 2018-02-15

**Authors:** Nan Dong, Ming-Ming Tang, Wei-Ping Zhang, Xing-Guo Bao, Yu Wang, Peter Christie, Long Li

**Affiliations:** 1 Beijing Key Laboratory of Biodiversity and Organic Farming, Key Laboratory of Plant-Soil Interactions, Ministry of Education, College of Resources and Environmental Sciences, China Agricultural University, Beijing, 100193 China; 2 Institute of Soils, Fertilizers and Water-saving Agriculture, Gansu Academy of Agricultural Sciences, Lanzhou, 730070 China

## Abstract

Intercropping studies usually focus on yield advantage and interspecific interactions but few quantify temporal niche differentiation and its relationship with intercropping yield advantage. A field experiment conducted in northwest China in 2013 and 2014 examined four intercropping systems (oilseed rape/maize, oilseed rape/soybean, potato/maize, and soybean/potato) and the corresponding monocultures. Total dry matter data collected every 20 d after maize emergence were fitted to logistic models to investigate the temporal dynamics of crop growth and interspecific interactions. All four intercropping systems showed significant yield advantages. Temporal niche complementarity between intercropped species was due to differences in sowing and harvesting dates or the time taken to reach maximum daily growth rate or both. Interspecific interactions between intercropped species amplified temporal niche differentiation as indicated by postponement of the time taken to reach maximum daily growth rate of late-maturing crops (i.e. 21 to 41 days in maize associated with oilseed rape or potato). Growth trajectories of intercropped maize or soybean recovered after the oilseed rape harvest to the same values as in their monoculture on a per plant basis. Amplified niche differentiation between crop species depends on the identity of neighboring species whose relative growth rate is crucial in determining the differentiation.

## Introduction

Nature has introduced great biodiversity into the world but humans have displayed a passion for simplifying it. Monoculture does not take advantage of the principles by which natural systems function, but instead represents agriculture from the perspective of an engineer^1^. Although farming systems have succeeded in supplying enough food for the majority of the global population, it is widely recognized that many of the systems based on sole cropping with substantial inputs of chemical fertilizers, pesticides, and antibiotics may have led to negative outcomes and vulnerabilities of agricultural ecosystems^2,3^. In contrast, intercropping, the simultaneous growth of two (or more) crop species in the same field area for all or part of their growing period (co-growth)^4,5^, has certain advantages over sole cropping. It is part of nature-based solutions in land management for enhancing ecosystem services^6^. Intercropping has been widely adopted by farmers in developing countries^7–11^, especially in the single-season cropping areas because of the annual thermal limitation in most areas of northwestern China^12,13^.

The main advantage of intercropping is the increase in productivity^14,15^ by exploiting the full duration of solar radiation^16–18^, thermal energy^19^, water^7,20,21^ and nutrient resources^4,22^ in resource-limited ecosystems. Moreover, intercropping can maintain or enhance soil quality^13,23^, promote biodiversity^3,24^, control weed growth^25^, minimize the incidence of pests and diseases^26^, reduce soil erosion and runoff discharge^6^, and increase farming incomes^5,8^. To design an intercropping system with advantages in terms of efficient resource utilization it is necessary to identify the relationship between the intercropping advantages and the growth traits and resource requirements of the component crop species^3^. Previous studies show that intercropping advantages depend greatly on niche differentiation in time^12,13^ and space^18,22,27,28^, or on positive interspecific interactions (facilitation)^17,23^ between intercropped species, thereby moderating competition^29^.

Plant competition has been studied for many decades but it is usually evaluated as a difference in biomass at a single, arbitrary, stage of growth^30^. The determination of the end-point harvest of biomass production can lead to inaccurate interpretations^31^. Furthermore, the same final patterns often result from different processes of competition^32^. For instance, in wheat/maize and wheat/soybean intercropping the ‘final’ biomass of intercropped maize or soybean is close to or significantly higher than that of sole crops at maturity. However, the neighbors (wheat) suppress the growth of intercropped maize or soybean at early growth stages^22,29^. Divergences in the biomass trajectories between intercropped and sole-cropped maize may therefore lead to a reliable interpretation^33^. Characterizing the development of plant–plant interactions during crop growth may help us to understand the potential performance of interspecific interactions^17,34^. Recent studies clearly show that the temporal dynamics of competition can be characterized using the logistic growth model^30,33,35–37^. Here, we employ the logistic growth function to analyze the growth trajectories of the interacting species in four intercropping systems with large variation in crop growth traits.

Oilseed rape, soybean, potato and maize have been widely grown in northwest China^15,20^. In addition, the four crops are different in growth traits such as stature^38,39^, root length and growth space^40^, and root distribution^41,42^. Spatial niche complementarity between crops may account for some of the overyielding of oilseed rape/maize, oilseed rape/soybean, potato/maize, and soybean/potato intercropping^27,39,40^. Previous studies have mostly investigated individual intercropping systems but few investigations have focused on multiple intercropping within the same study.

The objectives of the present study were to assess the yield advantage, the crop growth trajectories and the interspecific competitive dynamics in four intercropping systems, particularly in associated crops with large variation in traits such as growth pattern, growth season and plant morphology. It is important to understand the dynamics and mechanisms of competitive interactions and helpful to promote the adoption of novel intercropping systems if we are to maximize the advantages of intercropping in commercial practice.

## Results

### Intercropping advantages in grain yield and aboveground dry weight

Land equivalent ratios were 1.09–1.95 based on grain yields and were 1.02–1.63 based on aboveground dry weight in all four intercropping systems across the two years of the study (Table 1). The grain yields of oilseed rape/maize, oilseed rape/soybean, potato/maize and soybean/potato intercropping averaged over the two years increased dramatically by 12.6, 78.5, 15.1, and 21.7%, respectively. The above-ground dry weights increased by 15.8, 47.1, 21.6 and 40.8% compared with the corresponding expected values (Table 1).

**Table 1:** Observed or expected total grain yields, above-ground dry weight and land equivalent ratios (LER) in different intercropping systems in 2013 and 2014.

Combination	Year	Grain yield (t ha^−1^)			Above-ground dry weight (t ha^−1^)		
		Expected	Observed	LER	Expected	Observed	LER
Oilseed rape/maize	2013	9.6 ± 0.3^b^	12.0 ± 0.6^a^	1.76	19.0 ± 1.3^a^	23.0 ± 1.7^a^	1.32
	2014	10.6 ± 0.2^a^	10.7 ± 0.3^a^	1.32	22.2 ± 1.1^a^	24.7 ± 1.2^a^	1.3
	Mean	10.1 ± 0.4^B^	11.3 ± 0.6 ^A^	1.54	20.6 ± 1.5^B^	23.8 ± 1.4 ^A^	1.31
Oilseed rape/soybean	2013	1.7 ± 0.1^b^	3.3 ± 0.1^a^	1.95	6.4 ± 0.2^b^	9.8 ± 0.5^a^	1.55
	2014	2.5 ± 0.1^b^	4.2 ± 0.2^a^	1.65	9.0 ± 0.4^b^	12.8 ± 0.5^a^	1.45
	Mean	2.1 ± 0.3^B^	3.8 ± 0.3 ^A^	1.8	7.7 ± 0.9^B^	11.3 ± 1.1 ^A^	1.5
Potato/maize	2013	13.7 ± 0.4^a^	15.4 ± 0.7^a^	1.09	19.7 ± 0.2^b^	26.6 ± 0.4^a^	1.29
	2014	12.7 ± 0.2^b^	14.9 ± 0.2^a^	1.13	20.9 ± 0.7^a^	22.8 ± 1.2^a^	1.02
	Mean	13.2 ± 0.4^B^	15.2 ± 0.5 ^A^	1.11	20.3 ± 0.6^B^	24.7 ± 1.4 ^A^	1.16
Soybean/potato	2013	7.7 ± 0.6^a^	9.1 ± 0.1^a^	1.36	10.1 ± 0.7^b^	13.3 ± 0.6^a^	1.42
	2014	7.1 ± 0.1^b^	8.8 ± 0.3^a^	1.55	9.8 ± 0.1^b^	14.6 ± 0.7^a^	1.63
	Mean	7.4 ± 0.4^B^	9.0 ± 0.2 ^A^	1.46	9.9 ± 0.5^B^	14.0 ± 0.7 ^A^	1.53

NB: values (mean ± SE, *n* = 3) followed by the same lowercase letters for one crop combination are not significantly different between expected and observed grain yields or aboveground dry weight (horizontal comparison) within each year at the 5% level by LSD; values (mean ± SE, *n* = 6) with the same capital letter within each row within one crop combination are not significantly different between average expected and observed grain or aboveground dry weight across two years (one-way ANOVA, *P* < 0.05).

### Dynamic trajectories of above-ground dry weight in oilseed rape/maize intercropping

**Figure 1:**
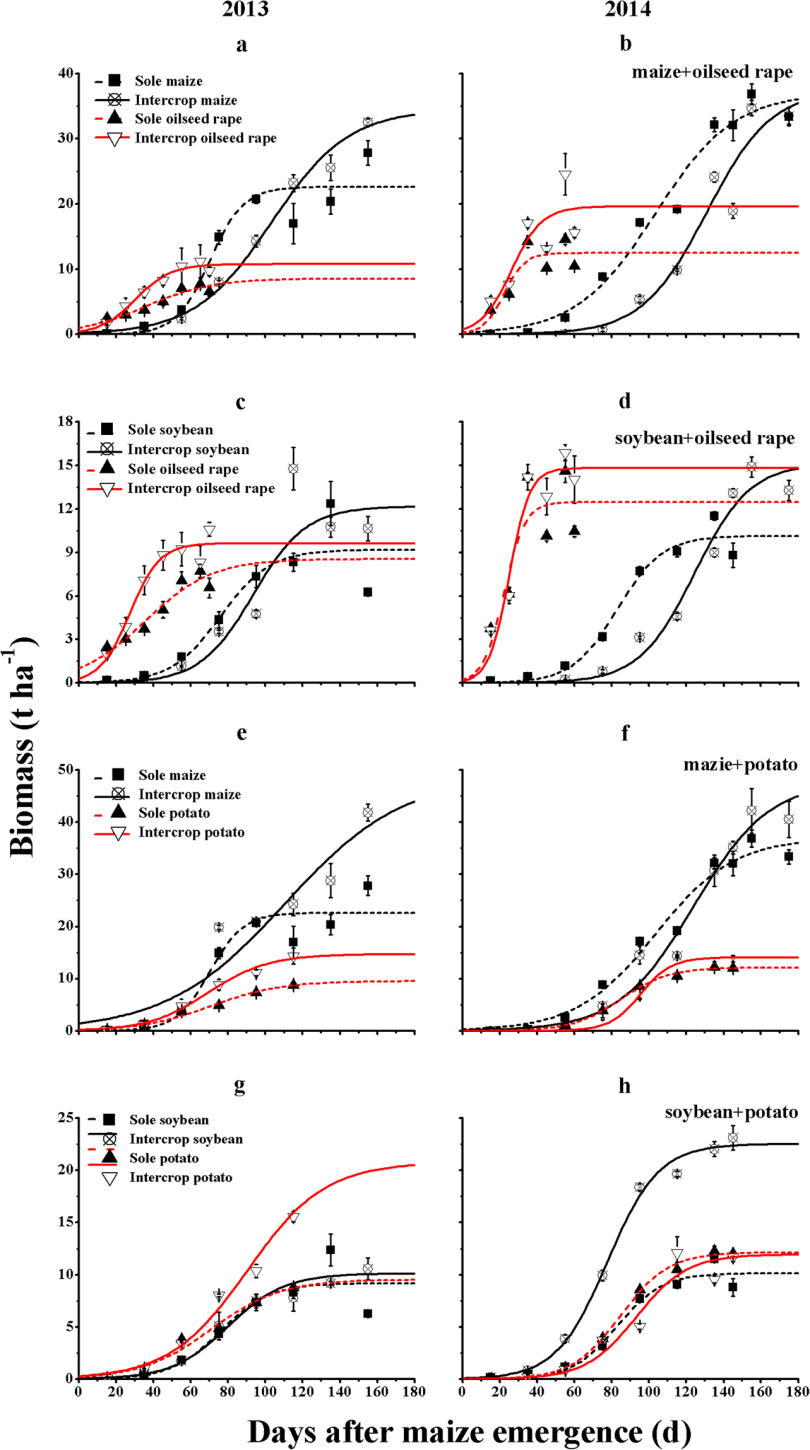
Dynamic cumulative biomass production of crops in different monoculture and intercropping systems in 2013–2014. All values represent mean ± SE (*n* = 3). Continuous curves denote intercropping, dashed curves denote sole crops, and the same color denotes one crop. The potato biomass was calculated by the dry matter of shoots plus tubers.

The cumulative dry matter of intercropped oilseed rape was higher than that of the monoculture and the maximum above-ground dry weight (*Y*_max_) of intercropped oilseed rape was close to that of sole oilseed rape in 2013 (Table 2, Fig. 1a) and significantly higher (by 57.0%) than that of sole cropping in 2014 (Table 2, Fig. 1b). In contrast, the above-ground dry weight of intercropped maize was significantly lower than that of sole maize before the oilseed rape harvest, but the maximum above-ground dry weight (*Y*_max_) of intercropped maize was significantly (52.6%) higher than that of sole maize in 2013 (Table 2, Fig. 1a).

**Table 2:** Logistic equation parameters for biomass (or whole potato biomass) accumulation in different intercropping systems in 2013 and 2014.

Intercropping pattern	Year	Treatment	*Y*_max_	*k*	*t*_max_	*I*_max_
			(t ha^−1^)	(×10^−3^ d^−1^)	(d)	(kg ha^−1^ d^−1^)
Oilseed rape/maize	2013	Sole maize	22.6 ± 2.8^b^	110 ± 26^a^	70 ± 5^b^	587 ± 94^a^
		Intercropped maize	34.5 ± 1.4^a^	48 ± 7^a^	103 ± 5^a^	414 ± 46^a^
		Sole oilseed rape	8.5 ± 0.7^a^	56 ± 4^b^	36 ± 3^a^	118 ± 1^b^
		Intercropped oilseed rape	10.8 ± 1.8^a^	105 ± 11^a^	30 ± 0^a^	272 ± 24^a^
	2014	Sole maize	37.0 ± 1.5^a^	47 ± 2^a^	103 ± 2^b^	435 ± 11^b^
		Intercropped maize	37.6 ± 2.4^a^	58 ± 3^a^	131 ± 3^a^	540 ± 17^a^
		Sole oilseed rape	12.5 ± 0.2^b^	180 ± 21^a^	22 ± 0^a^	563 ± 73^a^
		Intercropped oilseed rape	19.6 ± 1.3^a^	123 ± 24^a^	26 ± 2^a^	591 ± 87^a^
Oilseed rape/soybean	2013	Sole soybean	9.2 ± 0.6^b^	75 ± 4^a^	76 ± 3^b^	173 ± 15^a^
		Intercropped soybean	12.2 ± 0.4^a^	70 ± 4^a^	93 ± 2^a^	214 ± 9^a^
		Sole oilseed rape	8.5 ± 0.7^a^	56 ± 4^b^	36 ± 3^a^	118 ± 1^b^
		Intercropped oilseed rape	9.6 ± 0.8^a^	125 ± 11^a^	27 ± 2^a^	305 ± 54^a^
	2014	Sole soybean	10.1 ± 0.2^b^	83 ± 5^a^	83 ± 1^b^	210 ± 8^b^
		Intercropped soybean	15.1 ± 0.5^a^	67 ± 4^a^	124 ± 1^a^	255 ± 11^a^
		Sole oilseed rape	12.5 ± 0.2^a^	180 ± 21^a^	22 ± 0^a^	563 ± 73^a^
		Intercropped oilseed rape	14.8 ± 0.9^a^	184 ± 31^a^	25 ± 1^a^	677 ± 110^a^
Potato/maize	2013	Sole maize	22.6 ± 2.8^b^	110 ± 26^a^	70 ± 5^b^	587 ± 94^a^
		Intercropped maize	48.9 ± 3.2^a^	32 ± 3^b^	111 ± 8^a^	382 ± 32^a^
		Sole potato	9.5 ± 0.4^b^	53 ± 3^a^	71 ± 4^a^	127 ± 7^b^
		Intercropped potato	14.7 ± 1.5^a^	62 ± 4^a^	69 ± 3^a^	225 ± 13^a^
	2014	Sole maize	37.0 ± 1.5^b^	47 ± 2^a^	103 ± 2^b^	435 ± 11^a^
		Intercropped maize	47.9 ± 3.2^a^	49 ± 7^a^	124 ± 5^a^	580 ± 60^a^
		Sole potato	12.1 ± 0.4^b^	78 ± 7^b^	85 ± 1^b^	235 ± 18^b^
		Intercropped potato	14.1 ± 0.2^a^	121 ± 10^a^	95 ± 0^a^	426 ± 31^a^
Soybean/Potato	2013	Sole soybean	9.2 ± 0.6^a^	75 ± 4^a^	76 ± 3^a^	173 ± 15^a^
		Intercropped soybean	10.1 ± 0.9^a^	66 ± 15^a^	80 ± 5^a^	162 ± 29^a^
		Sole potato	9.5 ± 0.4^b^	53 ± 3^a^	71 ± 4^a^	127 ± 7^b^
		Intercropped potato	20.8 ± 3.2^a^	48 ± 6^a^	90 ± 8^a^	240 ± 9^a^
	2014	Sole soybean	10.1 ± 0.2^b^	83 ± 5^a^	83 ± 1^a^	210 ± 8^b^
		Intercropped soybean	22.5 ± 0.6^a^	73 ± 7^a^	78 ± 1^b^	411 ± 31^a^
		Sole potato	12.1 ± 0.4^a^	78 ± 7^a^	85 ± 1^a^	235 ± 18^a^
		Intercropped potato	11.9 ± 0.1^a^	73 ± 13^a^	93 ± 3^a^	218 ± 38^a^

NB: values (mean ± SE, *n = *3) followed by different small letters are significantly different between intercropping and sole cropping at the 5% level by LSD test.

**Figure 2:**
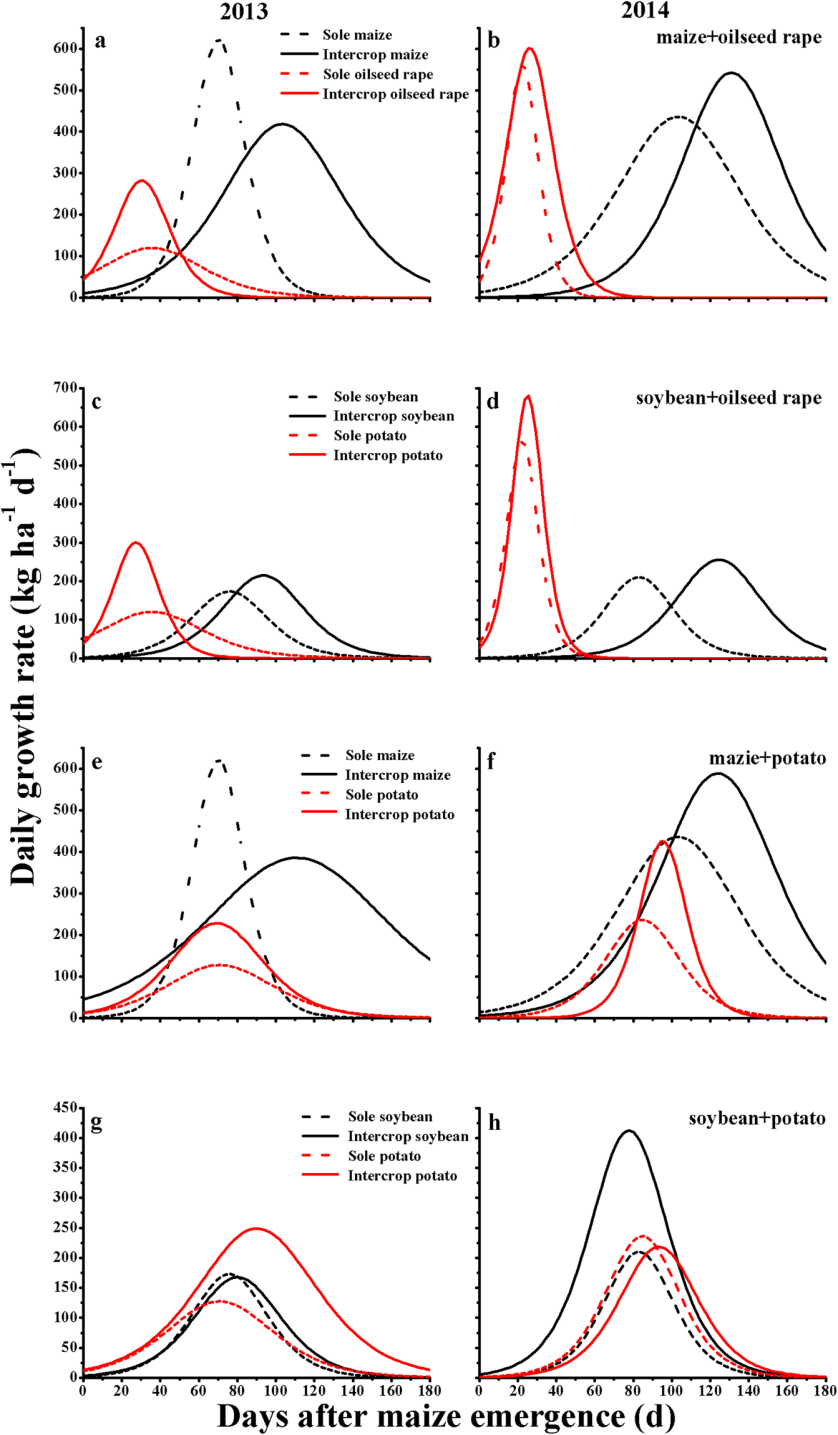
Daily growth rate (kg ha^−1^ d^−1^) of crops in different monoculture and intercropping systems in 2013–2014. Continuous curves denote intercropping, dashed curves denote sole crops, and the same color denotes one crop. Daily growth rate for potato is based on the whole potato individuals (shoots + tubers).

Sole maize and oilseed rape attained their maximum daily growth rates at 87 and 29 days, respectively, after maize emergence. Intercropped maize took significantly more days (an extra 28–33 days) to achieve its maximum daily growth rate than did monocultured maize. There was no significant delay in the above-ground dry weight of intercropped oilseed rape (Table 2; Fig. 2a,b).

Cropping system did not significantly affect the relative growth rate (*k*) of maize but intercropped maize had a higher maximum daily growth rate (*I*_max_; 540 kg ha^−1^ d^−1^) than sole maize (435 kg ha^−1^ d^−1^) in 2014 (Table 2). Intercropped oilseed rape was higher in both relative growth rate (*k*) and maximum daily growth rate (*I*_max_) compared to the corresponding monocultures in 2013, by 87.0 and 130.5%, respectively (Table 2, Fig. 2a) but there was no significant effect in 2014.

### Dynamic trajectories of above-ground dry weight in oilseed rape/soybean intercropping

The growth dynamic trajectories of all components were similar in different treatments in both years (Fig. 1). The maximum above-ground dry weight (*Y*_max_) of intercropped oilseed rape was close to that of sole oilseed rape (Table 2). In contrast, the above-ground dry matter of intercropped soybean was significantly lower than that of sole soybean during the co-growth stages. After the oilseed rape harvest the above-ground dry matter increased sharply and the maximum above-ground dry weight (*Y*_max_) of intercropped soybean increased significantly by 32.5 and 49.3% compared with sole soybean in 2013 and 2014, respectively (Table 2).

The times of maximum daily growth rate in sole soybean and oilseed rape were at 80 and 29 days after maize emergence, respectively. Intercropped soybean delayed the time taken to attain maximum daily growth rate (by 17–41 days) compared with sole soybean (Table 2; Fig. 2c,d). In contrast, there was no significant effect on the growth of oilseed rape.

Taking the whole cropping system into consideration, the relative growth rate (*k*) of intercropping was similar to that of sole cropping, but oilseed rape behaved differently in 2013 (Table 2; Fig. 2c,d). Again, intercropping enhanced the maximum daily growth rates (*I*_max_) by 158.5% compared to sole oilseed rape in 2013 (Table 2, Fig. 2c) and by 21.4% compared to sole soybean in 2014 (Table 2, Fig. 2d).

### Dynamic trajectories of above-ground dry weight in potato/maize intercropping

In both years the trajectories of all intercropped components were initially similar to those of the corresponding sole cropped species (Fig. 1e,f). However, the maximum above-ground dry weight (*Y*_max_) of both component species in intercropping systems increased by 15.7–116.3% compared with sole cropping (Table 2; Fig. 1e,f).

The time taken to reach the maximum daily growth rate (*t*_max_) of intercropped maize occurred 21–41 days (significantly) later than in sole maize (Table 2; Fig. 2e,f). Intercropping potato significantly postponed (by 10 days) the time taken to reach maximum daily growth rate (*t*_max_) compared to sole potato in 2014 (Table 2, Fig. 2f), but there was no significant effect in 2013 (Table 2, Fig. 2e).

Averaged over 2013 and 2014, there was no difference in relative growth rate (*k*) between intercropped and sole crops (Table 2). Subsequently, the maximum daily growth rate (*I*_max_) of intercropped potato was 77.2 and 81.3% higher than that of sole potato in 2013 and 2014, respectively (Table 2; Fig. 2e,f) but there was no significant effect on that of maize in either year.

### Dynamic trajectories of above-ground dry weight in soybean/potato intercropping

The growth dynamic trajectories of sole and intercropped soybean and potato were different in both years (Fig. 1g,h). In 2013 the growth trajectory of intercropped potato was greater than that of sole potato over the whole growing season but there was no significant difference between sole and intercropped soybean in above-ground dry weight (Fig. 1g). The maximum above-ground dry weight (*Y*_max_) of intercropped potato was 118.0% higher than that of sole potato, but there was no significant effect on that of soybean (Table 2, Fig. 1g). In 2014 the divergences of the above-ground dry weight curves of sole-cropped and intercropped soybean were significant over the whole growing season. However, the presence of a neighbor had no effect on the growth trajectories of potato (Fig. 1h). Subsequently, the maximum above-ground dry weight (*Y*_max_) of intercropped soybean was 122.1% higher than sole soybean, but there was no significant difference between sole-cropped and intercropped potato in maximum above-ground dry weight (*Y*_max_) (Table 2, Fig. 1h).

Similarly, the maximum daily growth rates (*I*_max_) of intercropped potato (2013) and soybean (2014) increased markedly by 89.0 and 95.7%, respectively, compared to the corresponding monocultures (Table 2; Fig. 2g,h).

Cropping system had no significant effect on either the relative growth rate (*k*) or the time taken to reach the maximum daily growth rate (*t*_max_) of the plants (Table 2; Fig. 2g,h) except for the *t*_max_ of intercropped soybean which was 5 days shorter than that of sole soybean in 2014 (Table 2, Fig. 2h).

## Discussion

Our study provides evidence that grain yields, above-ground dry weight and the maximum above-ground dry weights (*Y*_max_) of all intercropped species were approximately equal to or significantly higher than those of the corresponding monocultures (Tables 1, 2). Furthermore, we found that land use efficiency, measured as LER, was >1 in all four intercropping systems during the two years (Table 1). Similar yield advantages have long been recognized in other intercropping systems^5,24^ and a meta-analysis found that the average value of LERs was 1.22 ± 0.02^43^. Some previous studies have also demonstrated significant yield increases in oilseed rape/maize in the same area as our experiment^38,40^, and in oilseed rape/soybean in North America^9^, potato/maize in West Asia^10^ and common bean/potato in Africa^11^.

**Figure 3:**
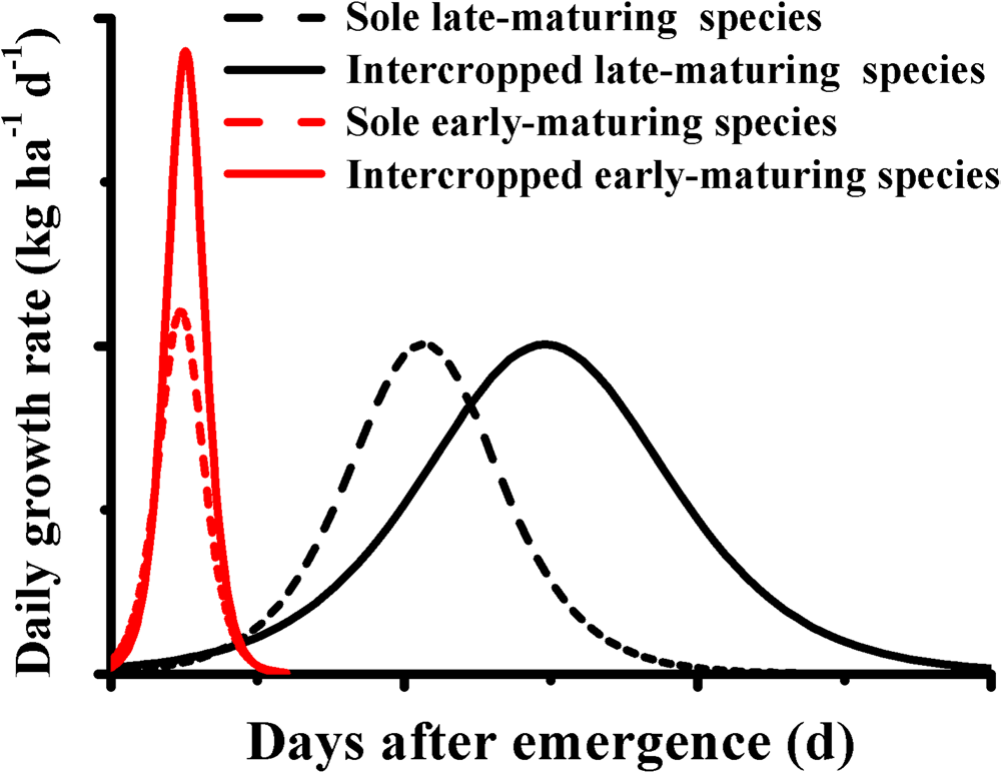
Schematic representation of mechanisms underlying temporal niche differentiation in intercropping systems.

In early/late-maturing species mixtures, e.g. oilseed rape/maize, oilseed rape/soybean and potato/maize, sowing and harvesting date were different from component crops (Table 3). These findings suggest the separation of growth periods between intercropped components and this may induce yield advantage in intercropping^25,43^. In addition, our study indicates that the time taken to attain maximum daily growth rate (*t*_max_) was also different between intercropped species in the three intercropping systems (Table 2; Fig. 2). Therefore, intercropped species obtained the resources at different times (as indicated by dashed lines in the conceptual model of Fig. 3). Niche differentiation refers to the process by which competitive species use the resources differently in time or space, which reduces interspecific competition and maintains species coexistence and complementarity in resource use by the various species^44^. Thus, our results suggest that ‘temporal niche differentiation’ indicated by the maximum daily growth rates (*I*_max_) by neighboring plant species is a key ecological mechanism in overyielding. More specifically, intercropping allowed plants to exploit the length of the growing season adequately^5,15^ and utilize available resources efficiently at separate times^17,18^.

**Table 3:** Crop management and sowing ratio of cropping systems and time of sowing and harvest in the field in 2013 and 2014.

Intercropping pattern	Year	Crop	Rows	Strip width (cm)	Crop width (cm)	Row distance (cm)	Gap between crops (cm)	Occupied proportion (%)	Sowing date	Harvest date
Oilseed rape/maize	2013	Maize	2	120	70	40	20	58	17 April	4 October
		Oilseed rape	5		50	10		42	27 March	10 July
	2014	Maize	2	120	80	40	25	67	21 April	15 October
		Oilseed rape	4		40	10		33	19 March	24 June
Oilseed rape/soybean	2013	Soybean	3	120	70	20	20	58	19 April	4 October
		Oilseed rape	5		50	10		42	27 March	10 July
	2014	Soybean	2	80	40	20	15	50	21 April	15 October
		Oilseed rape	4		40	10		50	19 March	24 June
Potato/maize	2013	Maize	1	90	30	40	50	33	17 April	4 October
		Potato	2		60	20		67	17 April	25 August
	2014	Maize	2	140	80	40	40	57	21 April	15 October
		Potato	2		60	20		43	21 April	19 September
Soybean/potato	2013	Soybean	2	90	40	20	25	44	17 April	4 October
		Potato	2		50	20		56	17 April	25 August
	2014	Soybean	2	100	40	20	30	40	21 April	19 September
		Potato	2		60	20		60	21 April	19 September

Our study therefore highlights significant postponement of the time taken to reach the maximum daily above-ground dry weight rate (*t*_max_) by 20–40 days by the later maturing intercropped species, e.g. maize in oilseed rape/maize and potato/maize or soybean in oilseed rape/soybean, compared with the corresponding monocultures (Table 2, Fig. 2). In the present study we find that intercropping strengthened temporal niche differentiation as indicated by the conceptual model in Fig. 3. The results of this study are in agreement with previous studies on the dynamic processes of above-ground dry weight accumulation^33,35–37^ and nutrient uptake^13^. Previous research suggests that crops reach their maximum daily growth rate at the stages of canopy closure and maximum leaf area^45^. Intense competition from neighboring plants usually decreases the survival, growth or reproduction of weak competitors^46^. It is therefore possible that prolonging the root lifespan and slowing down shoot senescence of intercropped maize or soybean may partly explain the delayed time of maximum growth rate^40,47^.

We also found that the dynamic trajectories of cumulative dry weight in oilseed rape/maize and oilseed rape/soybean intercropping systems can be explained in terms of the “competition-recovery production principle” of intercropping^22,29^. The trajectories of cumulative dry weight by intercropped oilseed rape clearly diverged from those of the monocultures at the early stages of the experiment, but the growth of intercropped maize or soybean was impaired before the oilseed rape harvest (Fig. 1a–d). In previous studies, intense competition occurred in early/late-maturing mixtures, and only early-maturing crops benefited from intercropping during the co-growth period^13,22,29,33^. This is the most likely explanation for the yield advantage of early-maturing crops in intercropping. After the oilseed rape harvest the trajectories of cumulative dry weight by intercropped maize or soybean increased sharply, and thereby the maximum above-ground dry weights (*Y*_max_) of intercropped soybean or maize were approximately equal to or significantly higher than those of the corresponding monocultures at maturity (Table 2, Fig. 1). The biomass production of late-maturing crops (soybean or maize) can effectively recover after the harvest of early-maturing crops (oilseed rape). Numerous studies have attributed such recovery or overyielding of growth by late-maturing crops to both the longer growing season^13,22,29,33^ and also the greater above- and below-ground space^15,39,47,48^.

Yield advantages were obtained in potato/soybean intercropping (Tables 1, 2), and the component species even shared a similar temporal niche by the absence of a significant difference in sowing dates, harvesting dates and the time taken to reach the maximum daily growth rate (*t*_max_) between both species (Tables 2, 3; Fig. 2g,h). In this experimental field, intercropped potato was planted in ridges and companion soybean was in furrows, thus the component species were inherently different in rooting pattern^41,42^ and shoot architecture^11^. The combination may improve their rhizosphere and canopy micro-environments due to the different spatial distributions of species relative to each other^18,27,39^. Previous studies show that the yield advantage of combinations containing legume species may be attributable to interspecific facilitation by processes such as N_2_ fixation^34,49^, N transfer^50^ and increased resource availability^23,24^.

The present study found that crop species can respond differently to various neighboring species. Soybean postponed the time taken to reach the maximum daily growth rate (*t*_max_) by 17–41 days when growing with neighboring oilseed rape, a fast growing species with 136 × 10^−3^ d^−1^ of average *k*, and did not delay the time when associated with potato, a species with a relatively slow growth rate with 63 × 10^−3^ d^−1^ of average *k* (Table 2, Fig. 2). Similarly, previous studies show that crop species had a substantially different growth pattern in two intercropping systems^28,49^. Interspecific facilitation between faba bean and maize enhanced nutrient uptake by maize, and the roots of intercropped maize spread underneath the faba bean plants^28,40^. However, competition from wheat resulted in a decrease in nutrient uptake by maize and limited the lateral spread of the roots of intercropped maize^28,48^. This highlights the importance of plasticity of crop response to different neighboring species in the design of new intercropping combinations.

**Figure 4:**
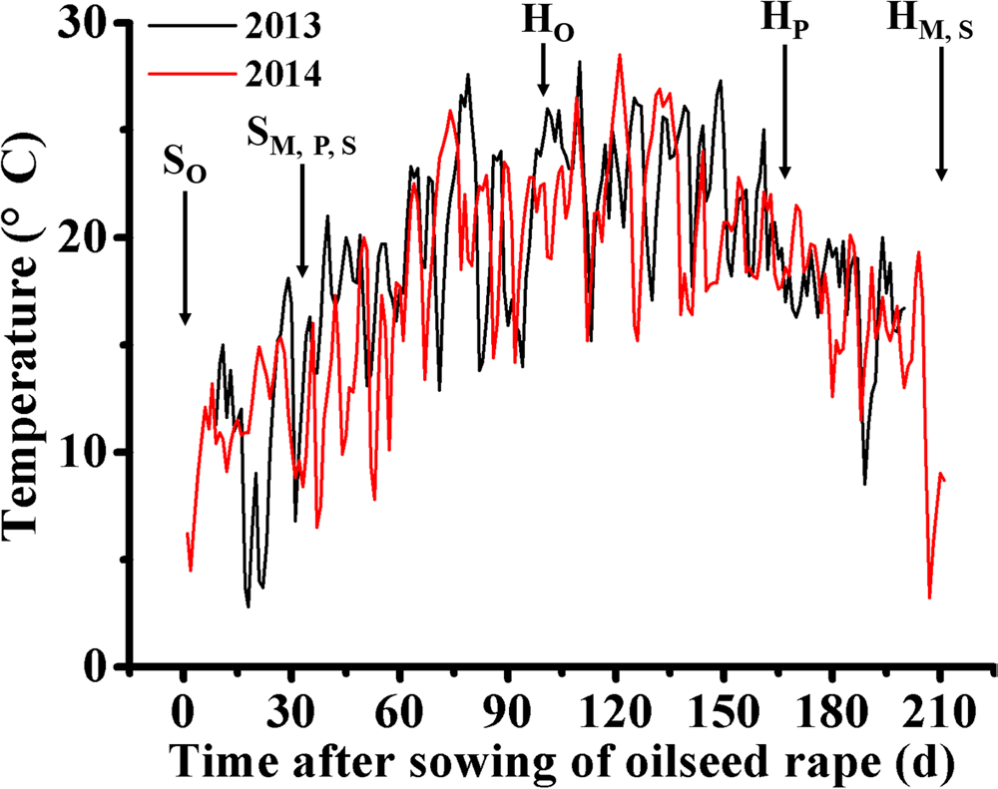
Mean daily temperatures (°C) in Wuwei during the two experimental periods. Time axis is days after sowing of oilseed rape. S_o_ = days of oilseed rape sowing, S_M, P, S_ = days of maize, potato and soybean sowing, H_o_ = days of oilseed rape harvest, H_P_ = days of potato harvest, H_M, S_ = days of maize and soybean harvest.

Our experiment indicates higher recovery of maize intercropped with oilseed rape in 2013 than in 2014 (Table 2; Fig. 1a,b), and in the soybean/potato intercropping system divergences in the dry weight trajectories between intercropped and sole plants were detectable in only one species at later growth stages in both years (Table 2; Fig. 1g,h). This may be due to the different row arrangements (Table 3, Fig. S1) in 2013 and 2014. Intercropping experiments on plant spacing have shown that the density of the component crops influences the interception of light^16,17^ and the total yield^7,45^. Furthermore, seasonal weather conditions, e.g. temperatures (Fig. 4), partially account for the differences in the results between years^19,51^.

## Materials

### Experimental site

The field experiments were conducted in 2013 and 2014 during the crop growing seasons (March-October) at the Baiyun Experimental Station, Wuwei, Gansu province, northwest China. The experimental site (38°37′N, 102°40′E) is 1504 m above sea level. Annual mean temperature is 10 °C. Cumulative temperatures above 0 °C and 10 °C are 4120 °C and 1729 °C, respectively. The duration of sunlight is 3002 h and total solar radiation is 5988 MJ m^−2^ year^−1^. The rainfall amounts to 161 mm. The frost-free period is 170–180 days. Air temperature trends during the experimental period in each year are presented in Fig. 4.

All experimental fields were used previously to grow sole maize. The 2014 experiment was conducted in a different field near the 2013 experimental field. The soil type is an Orthic Anthrosol^52^. The soil pH (in water) prior to sowing in 2013 was 7.62 and the soil contained 24 g kg^−1^ organic matter, 1.3 g kg^−1^ total N, 24.2 mg kg^−1^ Olsen-P, and 165.7 mg kg^−1^ exchangeable K per kilogram of dry soil in the top 20 cm of the soil profile. Similarly, soil organic matter was 21.4 g kg^−1^, total N 1.08 g kg^−1^, Olsen-P 23.6 mg kg^−1^, exchangeable K 69.5 mg kg^−1^ and pH (in water) 7.68 in 2014.

### Experimental design and crop management

Eight treatments comprising oilseed rape/maize, oilseed rape/soybean, potato/maize, soybean/potato, sole maize, sole oilseed rape, sole soybean, and sole potato were established each year with a randomized complete block design with three replicates. Maize (*Zea mays* L.) cultivar Xianyu No. 335, soybean (*Glycine max* L.) cultivar Fengdou No. 19, oilseed rape (*Brassica campestris* L.) cultivar Tianzhu, and potato (*Solanum tuberosum* L.) cultivar LK No. 99 are widely grown in northwest China.

Plot size was 3.6 × 6.0 m (4.2 × 6.0 m for potato/maize in 2014) and a total of 24 plots were established in an east-west row orientation. In oilseed rape/maize intercropping, 2 rows of maize alternating with 5 or 4 rows of oilseed rape were planted in each strip (Table 3; Fig. S1e,i). In oilseed rape/soybean intercropping, one strip was planted comprising 3 soybean rows and 5 oilseed rape rows in 2013, and 2 soybean rows and 4 oilseed rape rows in 2014 (Table 3; Fig. S1f,j). In potato/maize intercropping, one strip included 2 potato rows and 1 maize row in 2013, and 2 potato rows and 2 maize rows in 2014 (Table 3; Fig. S1g,k). Potato/soybean intercropping had 2 rows of potato alternating with 2 rows of soybean in each strip, but the width of each strip was different in 2013 and 2014 (Table 3; Fig. S1h,l). The sowing ratios of the cropping systems are shown in Table 3. The central strip of each plot remained free of damage until maturity in order to calculate grain yields. The remaining strips were used to collect samples continually during the growing season (the rows adjacent to the two ridges being discarded).

The inter-row and inter-plant distances of the cropping systems were the same in both sole and intercropped plots (Fig. S1). Soybean had two seeds in bunch planting. Oilseed rape was planted by broadcast sowing in each row. Maize and potato were planted mulched with white plastic film (0.90 m width). Potatoes were in ridges, and each ridge was 0.50 m high × 0.60 m wide.

Oilseed rape were sown in late March and harvested in late June or early July each year. Maize and potato were sown in late April with harvest dates in mid-October (maize) and late August to early September (potato) each year. Sole soybean and soybean intercropped with potato were harvested in late September or early October each year, but the harvest date of soybean intercropped with oilseed rape was almost one month later than that of sole soybean in 2014 (Table 3).

The same rate of fertilizer nitrogen (165 kg N ha^−1^ as urea) was applied to potato, soybean and oilseed rape with double this rate applied to maize in both sole and intercropping systems. Before sowing, all the fertilizer P (120 kg P ha^−1^, applied as triple superphosphate) and 165 kg ha^−1^ of the fertilizer N were evenly broadcast into the top 20 cm of the soil profile, with two topdressings of the fertilizer N (82.5 kg ha^−1^ each) for intercropped and sole maize at the maize stem elongation stage and the pre-tasseling stage, respectively. No potassium (K), organic manure or fungicide was applied to any crop. All plots were weeded by hand.

All plots were adequately irrigated seven times throughout all growth stages to prevent water stress (Table 4). Each irrigation was 75 mm (750 m^3^ ha^−1^), and the irrigation practice was applied at the same times according to conventional local farming practice each year.

**Table 4:** Specific irrigation times for each treatment

Treatment	Irrigation dates						
	20–25 April	20 May	5–10 June	20–25 June	15 July	5 August	30 August
Sole oilseed rape	+	+	+				
Sole maize		+	+	+	+	+	
Sole potato		+	+	+	+		
Sole soybean		+	+	+	+	+	
Oilseed rape/maize	+	+	+	+	+	+	+
Oilseed rape/soybean	+	+	+	+	+	+	+
Potato/maize		+	+	+	+	+	
Soybean/potato		+	+	+	+	+	

NB: “+” is defined as the irrigation date.

### Plant sampling and analysis

The first samples of crops were taken 15 days after maize emergence. Ten plants of maize, soybean and potato were cut at ground level randomly due to the smaller straw at this time. Subsequently, four plants were sampled at 20-day intervals. When the tubers emerged, the shoots and tubers of potato were measured for calculation of the whole dry matter yields. Because oilseed rape is broadcast-sown and has a short growth period, each sample was taken once every 10 days. The sampling areas were 20 cm × 5 rows (2013) or 20 cm × 4 rows (2014).

At maturity, the sampling areas were 6 m × 2 rows for maize and potato, 6 m × 5 rows (2013) or 6 m × 4 rows (2014) for oilseed rape, and 6 m × 3 rows or 6 m × 2 rows for soybean. Maize, soybean and oilseed rape were divided into seeds and straw for calculation of grain yields and above-ground dry matter yields. The shoot and tuber dry weights of potato were determined.

Plant samples were oven dried at 105 °C for 30 min and then at 65 °C for 72 h to constant weight. Dry matter values determined during the cropping seasons were used to calculate the dynamic trajectories of crop growth.

### Calculations

The comparison between observed and expected yields^53^ was used to evaluate the overyielding of intercropping systems in grain yield and above-ground dry weight. The total grain yield in intercropping was calculated as:

1 $${Y}_{\exp }={Y}_{\exp ,a}+{Y}_{\exp ,b}={M}_{a}\times {P}_{a}+{M}_{b}\times {P}_{b}$$

where M_a_ and M_b_ are the grain yields (per unit of total area of the intercrop) of crops ‘a’ and ‘b’ in the sole cropping system. P_a_ and P_b_ are the proportions of the area occupied by the individual crop species in the intercropping system (Table 3). This expectation is based on the null hypothesis that the grain yield per individual plant is the same in intercropped and sole crops. If observed grain yields are greater than expected the grain yield per plant is greater in intercropping than in the sole crop. The above equations were also used to calculate the aboveground dry weight (per unit of total area of the intercrop). The grain yield of potato refers to tuber dry matter and the above-ground dry matter yield of potato includes shoots and tubers.

In all analyses the land equivalent ratio (LER) is generally used to evaluate the land use advantage of intercropping^2^ and is defined as follows:

2 $$LER=\frac{{Y}_{a}}{{M}_{a}}+\frac{{Y}_{b}}{{M}_{b}}$$

Where *Y*_a_ and *Y*_b_ are the above-ground dry weights or grain yields (per unit of total area of the intercrop) of intercropped species a and b. An intercropping system exhibits a land use advantage if LER >1.0 and conversely no yield advantage if LER <1.0^2^.

The logistic growth function using least squares has been used increasingly to fit above-ground dry weight yield data (at least six harvests) from emergence until death or harvest^30,37^. The logistic growth equation comprises:

3 $${M}_{t}=\frac{{Y}_{\max }}{1+{e}^{k({t}_{\max }-t)}}$$

Where *M*_t_ (kg ha^−1^) is the above-ground dry weight per unit ground area of each crop component grown in a given treatment at (*t*) days after maize emergence during the growing season. *Y*_max_ (kg ha^−1^) is a parameter determining the asymptotic maximum above-ground dry weight, *k* (d^−1^) is the relative growth rate (d*M*_*t*_/d*t* × 1/*M*_*t*_), and *t*_max_ (d) is the time taken to reach maximum daily growth rate (d*M*_*t*_/d*t*). These parameters were determined using the Slogistic1 procedure of the OriginPro8 software (OriginLab Corporation, Northampton, MA).

The daily growth rate^13,33^ is:

4 $$\frac{{\rm{d}}{M}_{t}}{dt}=k{M}_{t}(1-\frac{{M}_{t}}{{{\rm{Y}}}_{{\rm{\max }}}})$$

The daily growth rate attains a maximum at *M*_*t*_ = *Y*_max_/2, therefore the maximum daily growth rate, *I*_max_ = *k Y*_max_/4, occurs at time *t*_max_.

### Statistical analysis

The main effects of the cropping treatments on the four parameters (*k*, *Y*_max_, *t*_max_ and *I*_max_) were determined using analysis of variance (ANOVA). One-way ANOVA was used to compare the significance of differences among all treatments and pairs of treatment mean values were compared using least significance difference (LSD) at the 5% level. All statistical analysis was carried out using the SAS version 8.2 software package (SAS Institute, 2003).

## Electronic supplementary material

Figure S1
