# Supplementary material for: Temporal Differentiation of Crop Growth as One of the Drivers of Intercropping Yield Advantage
Source: Sci Rep. 2018 Feb 15;8:3110. doi: 10.1038/s41598-018-21414-w (PMC5814522; doi:10.1038/s41598-018-21414-w)
Supplement: Supplementary file 1 — Figure S1 [file 41598_2018_21414_MOESM1_ESM.doc]

**Supporting information**

**Temporal Niche Differentiation as One of the Drivers of Intercropping Yield Advantage**

Nan Dong^1^, Ming-Ming Tang^1^, Wei-Ping Zhang^1^, Xing-Guo Bao^2^, Yu Wang^1^, Peter Christie^1^, Long Li^1^, *

^1^ Key Laboratory of Plant-Soil Interactions, Ministry of Education, Beijing Key Laboratory of Biodiversity and Organic Farming, College of Resources and Environmental Sciences, China Agricultural University, Beijing 100193, China.

^2^ Institute of Soil, Fertilizer and Water-saving Agriculture, Gansu Academy of Agricultural Sciences, Lanzhou 730070, China.

Correspondence and requests for materials should be addressed to L.L. (lilong@cau.edu.cn)


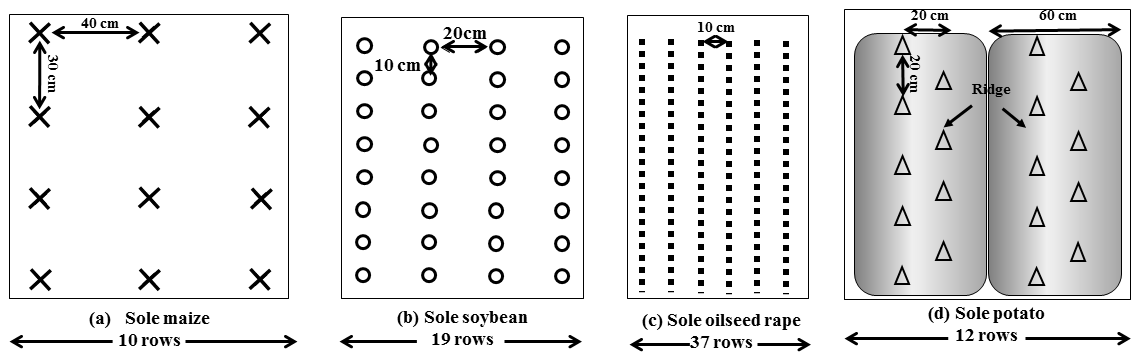


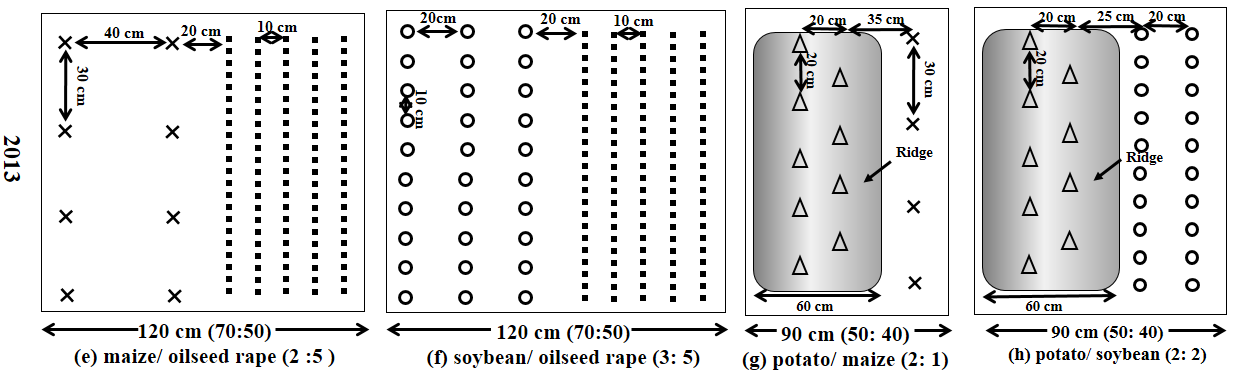


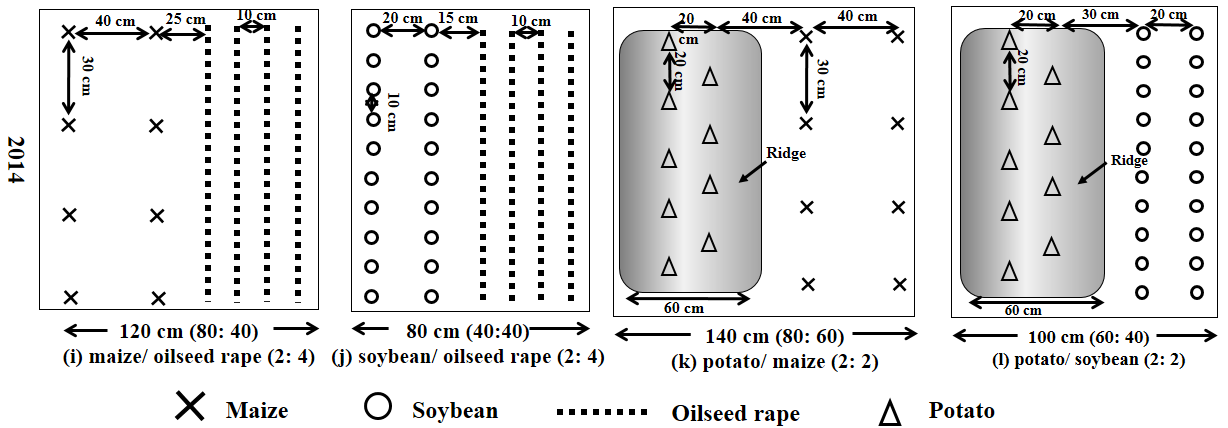


**Figure S1** Diagram showing the arrangement of the rows of maize (
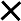
), soybean (), oilseed rape (--) and potato () in the field experiment. (a) sole maize; (b) sole soybean; (c) sole oilseed rape; (d) sole potato; (e, i) soybean/oilseed rape intercropping; (f, j) maize/oilseed rape intercropping; (g, k) potato/maize intercropping; (h, l) potato/soybean intercropping; (e, f, g and h) field arrangement of different cropping systems in 2013; (i, j, k and l) field arrangement of different cropping systems in 2014.
